# Interaction between mitochondria and microbiota modulating cellular metabolism in inflammatory bowel disease

**DOI:** 10.1007/s00109-023-02381-w

**Published:** 2023-10-11

**Authors:** Misa Hirose, Priyadharshini Sekar, Mariam Wed Abdelaziz Eladham, Mohammad T. Albataineh, Mohamed Rahmani, Saleh Mohamed Ibrahim

**Affiliations:** 1 Lübeck Institute of Experimental Dermatology, University of Lübeck, Ratzeburger Allee 160, 23562 Lübeck, Germany; 2 Sharjah Institute of Medical Research, RIMHS, University of Sharjah, Sharjah, United Arab Emirates; 3 College of Medicine and Health Sciences, Khalifa University, Abu Dhabi, United Arab Emirates

**Keywords:** Mitochondria-microbiota interaction, Inflammatory bowel disease (IBD), Cellular metabolism, Mitochondrial DNA (mtDNA) polymorphisms, Bacterial metabolites, Mitochondrial reactive oxygen species (ROS)

## Abstract

Inflammatory bowel disease (IBD) is a prototypic complex disease in the gastrointestinal tract that has been increasing in incidence and prevalence in recent decades. Although the precise pathophysiology of IBD remains to be elucidated, a large body of evidence suggests the critical roles of mitochondria and intestinal microbiota in the pathogenesis of IBD. In addition to their contributions to the disease, both mitochondria and gut microbes may interact with each other and modulate disease-causing cell activities. Therefore, we hypothesize that dissecting this unique interaction may help to identify novel pathways involved in IBD, which will further contribute to discovering new therapeutic approaches to the disease. As poorly treated IBD significantly affects the quality of life of patients and is associated with risks and complications, successful treatment is crucial. In this review, we stratify previously reported experimental and clinical observations of the role of mitochondria and intestinal microbiota in IBD. Additionally, we review the intercommunication between mitochondria, and the intestinal microbiome in patients with IBD is reviewed along with the potential mediators for these interactions. We specifically focus on their roles in cellular metabolism in intestinal epithelial cells and immune cells. To this end, we propose a potential therapeutic intervention strategy for IBD.

## Introduction

Inflammatory bowel disease (IBD) is a common complex disease in the gut, and its global incidence and prevalence have been increasing worldwide in recent decades [1]. IBD primarily includes two subtypes: Crohn’s disease (CD) and ulcerative colitis (UC). The pathophysiology of IBD is complex, involving genetic and environmental factors [2] and impaired intestinal barrier function and disruption of innate and adaptive immune responses [3, 4]. However, the precise underlying disease mechanisms are not yet fully understood, despite many scientific efforts. Current therapies for IBD include untargeted therapies, such as glucocorticoids, and targeted biologic therapies, which are effective for some but not all patients [2]. Therefore, identifying pathways and mediators involved in disease pathology is the key to discovering therapeutic targets and developing novel therapeutic options for IBD.

This review summarizes current knowledge about the pathological roles of the microbiome and mitochondria in chronic inflammatory diseases in the gut, namely IBD. Furthermore, we discuss the potential interaction between mitochondria and microbiota and their contribution to IBD pathology, particularly cellular metabolism. This concept may contribute to elucidating novel pathways involved in IBD and to the discovery of new therapeutic options for IBD as well as other chronic inflammatory diseases in the gut. Finally, we propose a potential approach to modulate such a mitochondria-microbiota axis thereby controlling the disease.

## Structure of the intestinal barrier at the cellular level

The gastrointestinal tract consists of four layers: mucosa, submucosa, muscular layer, and serosa [5]. The mucosa is further subdivided into three layers: a simple columnar epithelium, surface mucus layer, and underlying immune cell-containing lamina propria [6]. The epithelium consists of heterogenous cell types with its specialized functions. These include intestinal epithelial cells (enterocytes), goblet cells, Paneth cells, tuft cells, microfold (M) cells, enteroendocrine cells, and epithelial stem cells [6, 7]. Small and large intestine greatly differ in structure and cellular composition [8]. The small intestine comprises the duodenum, jejunum, and ileum, while the large intestine includes the cecum, colon, and rectum. The small intestinal epithelium forms crypts and has villi, and epithelial cell possesses striated microvilli. In contrast, the large intestine has crypts but lacks villi. Mucus covers the small and large intestine, and its layer is much thicker in the large intestine. The small intestine has Peyer’s patches, the main site of M cells. Intestinal epithelial cells serve as the intestinal barrier and are responsible for nutrient absorption, sensing pathogen-associated molecular patterns, secretion of antimicrobial peptides (AMPs), and modulation of immune cells. Goblet cells secrete mucus that forms an additional barrier of host protection and are more prevalent in the large intestine. Paneth cells secrete AMPs and growth factors for epithelial stem cells and are present in the small intestine [9]. M cells are specialized cells controlling the initiation of mucosal immune response by transporting antigens and microorganisms to the underlying lymphoid tissue [10]. Tuft cells monitor intestinal content, such as substances and intestinal pathogens using succinate, taste receptors, and respond to these by secreting cytokines and endocrine signaling molecules [11]. Enteroendocrine cells have multiple chemosensory receptors and able to detect intestinal microbes and microbe-derived metabolites. Upon these stimulations, enteroendocrine cells secrete peptide hormones and cytokines, thus modulate immune system [12]. As such, the intestinal epithelium plays an essential role in the selective absorption of nutrients and functions as a physical barrier separating mucosal tissues from the exterior environment, e.g., luminal commensal bacteria, pathogens, and dietary antigens.

Another important cell compartment in the intestinal mucosa is immune cells. These immune cells generally reside within one of three compartments: the epithelium (termed intraepithelial lymphocytes, which are primarily T cells with T-cell receptor γδ and T-cell receptor αβ lineages), the lamina propria (termed lamina propria lymphocytes, mainly CD4+ T cells, CD8+ T cells and plasma cells as well as dendritic cells, macrophages, mast cells, eosinophils, and innate lymphoid cells; ILCs), and specialized intestine-associated lymphoid structures in Peyer’s patches and mesenteric lymph nodes (including B cells, CD4+ T cells, CD8+ T cells, dendritic cells, and macrophages) [13]. These immune cells in the intestinal mucosa, together with intestinal epithelial cells, coordinate the mucosal homeostasis, which makes the host tolerant to foodborne antigens and commensal bacteria or develops a response against pathogenic bacteria. If this communication between immune cells and epithelial cells is dysregulated for any reasons, it will result in pathogenic mucosal inflammation. In fact, the disruption of the epithelial barrier and excessive immune response are hallmarks of IBD, as mentioned above. For example, intestinal T-cell infiltrate in IBD patients demonstrates higher levels of proinflammatory T cells and impaired activity of regulatory T cells compared with that in healthy controls and nonactive IBD tissue, and the subsets of monocytes and macrophages in the peripheral blood and colon tissue of IBD patients were significantly altered compared with those of healthy controls [14]. In the next sections, we will review factors that are thought to contribute to the dysregulation of these cells leading to IBD: gut microbiota and mitochondria.

## Evidence for the important role of the gut microbiota in IBD

### Association between dysbiosis and IBD

Due to the complex nature of pathological processes, multiple factors are thought to contribute to the disease pathology in complex diseases, including IBD. A number of genetic studies have been conducted and successfully identified over 200 genetic loci associated with IBD [15–18]. Moreover, over 70% of IBD cases cannot be explained only by genetics [15]. Another contributing factor to IBD is the environment, which includes diet lifestyle, air pollution such as particle matters and ozone, temperature (seasons), and climate changes [19, 20], a factor shared with other noncommunicable diseases [21, 22]. Considering the sharp increase in IBD incidence during recent decades, what has also largely changed is our lifestyle, including diet, antibiotic use, social life, and physical activities. In contrast, our genome has not been dramatically changed within the same time period. Importantly, such changes in our lifestyle can influence the composition of commensal bacteria in our gut [23].

Multiple studies have demonstrated that an imbalance of the gut microbial community, namely, dysbiosis, is associated with human diseases, including IBD [24]. Moreover, a direct causal effect of dysbiosis in IBD has not been proven in humans to date [25], while there are observations indicating the major role of intestinal microbiota in IBD. These observations include (1) clinical disease improvement after antibiotic treatment in CD patients [26, 27]; (2) the effectiveness of fecal transplantation in IBD patients, especially those suffering from UC [28–30]; and (3) the global increase in IBD associated with changes in lifestyle, such as diet and environment [31]. Nevertheless the outcome of antibiotics treatment efficacy was inconsistent between clinical trials [32, 33].

Classically, there is strong evidence of specific bacteria that are associated with pathological processes in IBD. These include adherent-invasive *Escherichia coli*, *Fusobacterium nucleatum*, *and Clostridium difficile* [34–36]. These bacterial species are known to be invasive or toxin-producing, thus have been implicated in pathogenesis of intestinal diseases [37]. In fact, IBD patients particularly under biologic and antibiotics treatment are at higher risk of *Clostridium difficile* infection, which was associated with relapse and higher mortality [38].

### Bacterial metabolites as disease mediators: secondary bile acids, SCFAs

Dysbiosis changes not only the composition of the intestinal microbiota but also its metabolites, which are likely involved in the pathogenesis of IBD [37, 39–41]. Such examples include sphingolipids, bile acids, and short-chain fatty acids (SCFAs), among others [39, 42, 43].

In a recent study of untargeted metabolomic and metagenomic profiling in two IBD cohorts, the levels of bile acids (cholate and chenodeoxycholate, thus primary bile acids) and sphingolipids in IBD patients were significantly higher than those in healthy individuals [39]. In relation to the increased levels of primary bile acids, a complementary decrease in secondary bile acids was observed in IBD patients in the same study because primary bile acids support the digestion of lipids and are deconjugated by microbes to secondary bile acids. The effect of secondary bile acids on intestinal epithelial cells was demonstrated by several experimental studies. Secondary bile acids (lithocholic acid and deoxycholic acid) exhibited anti-inflammatory effects on intestinal mucosa by inhibiting proinflammatory cytokine IL-1beta and IL-8 secretion in the Caco-2 human colon adenocarcinoma cell line [44] and promoted intestinal epithelial regeneration in mice [45].

Similar to secondary bile acids, significantly lower levels of SCFAs were identified in IBD patients than in healthy individuals [46]. SCFAs are also involved in epithelial barrier function. SCFAs include acetate, propionate, and butyrate, and these are the most abundantly produced by anaerobic fermentation of dietary fibers in the gut [43]. SCFAs promoted cell proliferation [47], epithelial barrier integrity [48], and the production of antimicrobial peptides, which are a class of peptides with inhibitory effects against pathogenic microbes [49], in intestinal epithelial cells in experimental settings. This experimental evidence indicates that bacteria-derived metabolites play a critical role not only in maintaining epithelial barrier function but also as first-line defense effectors against pathogens. In addition to such effects of bacterially derived metabolites on intestinal epithelial cells, both secondary bile acids and SCFAs exert modulatory functions in intestinal immune cells, which are relevant to IBD pathology. Intestinal macrophages, T regulatory cells, and effector T cells are known to be regulated by secondary bile acid metabolites [42]. Similarly, SCFAs were reported to regulate colonic T regulatory cells via the receptor GPR43 [50] and to promote anti-inflammatory effects in colonic macrophages and dendritic cells via the receptor GPR109a, promoting the differentiation of T regulatory cells and IL-10-producing T cells [51]. Furthermore, enema treatment with butyrate in UC patients significantly decreased the disease activity index accompanied by a reduction in NF-kappa B activation in lamina propria macrophages [52]. On the other hand, butyrate enema showed no or only minor effect on disease activity index, clinical symptoms, endoscopic and histological scores, and inflammatory markers in IBD patients in other clinical studies [53–55]. Application of butyrate was exclusively via enema, which is a safe approach, in all of these clinical studies. Therefore, evaluation of other administration strategies, such as intestinally targeted drug delivery system using a nanocapsulated form, may be a potential option to improve the clinical outcome of butyrate.

In summary, dysbiosis is associated with IBD and may be involved in the disease pathology by changing not only the composition of the intestinal microbiota (e.g., increased levels of pathobionts) but also the profile of gut microbe-derived metabolites, which impact the activities of intestinal epithelial cells and immune cell populations that contribute to IBD pathology. However, therapeutic application of such intestinal microbial metabolites in IBD still requires further reliable data from randomized controlled trials, despite the sufficient experimental evidence that such mediators have beneficial effects at cellular levels.

## Mitochondrial dysfunction is associated with IBD

### Mitochondrial dysfunction as etiology of IBD

Bacterial involvement in pathophysiology of IBD was discussed in the previous sections, as one of the environmental factors associated with IBD. Next, mitochondrial dysfunction, as a genetic factor, is discussed. Mitochondrial dysfunction is involved in pathogenesis of a wide range of diseases in multiple organs, including IBD. For example, genome wide association studies have identified over 200 candidate loci to date [16, 56, 57]. These include mitochondrial functionally related genes. For example, *ALDH2*, *LLRK2*, *and STAT3* were identified as genome-wide significantly associated genes with IBD [16, 58]; *SLC22A5*, *C13orf31*, *GPX1*, *and GPX4* were those with CD [56], and *PARK7* was that with UC [57]*.* These genes are relevant to cellular metabolism (*ALDH2*, *SLC22A5*, and *C13orf31*), apoptosis (*LLRK2*), redox sensing (*PARK7*), and redox balance (*GPX1* and *GPX4*). In addition, independent unbiased analysis of the data [16] with specific focus of mitochondrial ontology reveled that additional 22 genes involved in mitochondrial function within the subset of 574 out of total 22,353 genes within 100 kb of a genome-wide significant IBD locus [59]. These genes include those related with mitochondrial iron transport (*SLC25A28*, *VARS*, and *RNF5*), mitochondrial unfolded protein responses (*HSPA1-A*, *-B*, and -*L*), and mitochondrial oxidative phosphorylation machinery (*NDUFAF3*, *SDHC*, and *UQCR10*) [59]. As such, mutations in genes responsible for mitochondrial function are partly involved in pathogenesis of IBD.

### Mitochondrial function

Mitochondria are cellular organelles that process dietary nutrients into usable energy in the form of adenosine triphosphate (ATP) through oxidative phosphorylation (OxPhos) in serial enzyme complexes called the electron respiratory chain (ETC) in the inner membrane of the mitochondria. During the OxPhos reaction, mitochondria generate reactive oxygen species (ROS) as a byproduct [60]. Mitochondrial ROS control cell death [61] and are also major inflammatory mediators that activate the NLRP3 inflammasome [62]. In the mitochondrial matrix, there are enzymes that are involved in the tricarboxylic acid (TCA) cycle, fatty acid oxidation (FAO), amino acid oxidation, heme synthesis, and iron sulfur cluster formation [63]. Of note, the TCA cycle produces not only NADH^+^ and FADH_2_^+^, which fuel complex I and complex II in OxPhos but also generates intermediates such as acetyl coenzyme A (acetyl-CoA), alpha-ketoglutarate, and succinate. These metabolic intermediates function as signaling molecules and control cellular responses [63]. Therefore, mitochondria are called the cellular metabolic hub. In addition, mitochondria control Ca^2+^ homeostasis [64], epigenetic modification [65], and apoptosis [66]. The induction of mitochondria-driven apoptosis pathway is triggered by permeabilization of mitochondrial outer membrane. Upon diverse cellular stresses such as growth-factor deprivation and DNA damage, mitochondrial outer membrane permeabilization, caused by effector pro-apoptotic members of the B cell lymphoma 2 (BCL-2) family of proteins, such as BAX and BAK, mediates the leakage of soluble proteins (such as cytochrome c) from the mitochondrial intermembrane space to the cytosol. Cytochrome c released from the mitochondria subsequently binds to the cytosolic protein apoptotic protease-activating factor and forms apoptosome, which recruits and activates the initiator caspase 9, followed by cleavage and activation of caspase 3 and 7 resulting in cell death [66]. Similarly, the voltage-dependent anion channel 1 (VDAC1), a protein locating at the outer membrane of mitochondria, is also involved in the regulation of release of mitochondrial pro-apoptotic proteins, cytochrome c, and interacting with BCL-2 family proteins [67]. Since mitochondria are present in almost all cell types, functional deterioration of mitochondria affects the functions of any organ. In fact, mitochondrial dysfunctions are directly linked to common diseases, including neurodegenerative, metabolic, age-related, and chronic inflammatory disorders, including IBD [68–70].

### Mitochondrial function in intestinal epithelial cells and its pathological relevance to IBD

Intestinal epithelial cells consist of distinct cell types, including enterocytes, goblet cells, Paneth cells, and Lgr5^+^ crypt base columnar stem cells, as mentioned earlier. All intestinal epithelial cell types facilitate distinct metabolic profiles according to specialized function, thereby maintaining homeostasis in the gut [71]. Dysregulation of cellular metabolism in such fine-tuned cellular compartments alters intestinal functions and consequently results in disease involvement. In fact, there is evidence showing mitochondrial functional relevance in IBD patients, which will be presented in this section.

Mitochondria isolated from colon mucosa obtained from Brazilian UC patients showed mitochondrial respiratory chain complexes II, II, and IV were significantly decreased by around 50 to 60% compared with the mitochondria isolated from control colon mucosa [72]. Similarly, tissue homogenates of colon mucosal biopsy samples from Indian UC patients showed significant reduction of complex II activities compared with controls [73]. These studies suggest an involvement of mitochondrial dysfunction in pathogenesis of UC. A recent multicenter study conducting a bulk RNA-sequencing study of UC patients revealed a striking downregulation of genes involved in mitochondrial metabolism–associated genes and pathways in UC [74]. A proteomics analysis of UC-diseased colon mucosa demonstrated a significant reduction in mitochondrial proteins involved in energy production [75]. For CD, there is a case report suggesting a potential involvement of mitochondrial dysfunction (in complex III and IV) in the pathogenesis of CD [76]. Energy deficiency caused by mitochondrial dysfunction in intestinal cells is thought to link to the pathological consequence in both UC and CD. More specifically, energy deficiency in enterocytes leads to compromised nutrient absorption in the small intestine and water and electrolytes absorption in the colon [77]. Goblet cells with energy deficits result in insufficient mucus production in the colon [78]. Energy deficiency in Paneth cells impacts stem cell homeostasis and production of AMPs, resulting in compromised turnover of epithelial cells and dysbiosis in the small intestine, respectively [79]. These contribute to the ileal and colonic pathogenesis in CD and UC, respectively.

As mentioned earlier, mitochondria also control apoptosis. To keep intestinal homeostasis, continuous division of stem cells leads to generation of progenitor cells, which rapidly proliferate and transform into mature intestinal epithelial cells. Excessive apoptosis of differentiated intestinal epithelial cells will accelerate proliferation of immature crypt cells, which can damage the intestinal epithelial barrier. This will result in hyperpermeability and invasion of commensal or environmental microbes into the tissue, which are involved in pathology of IBD [80]. In fact, upregulated expression of VDAC1, which mediates mitochondria-driven apoptosis, was observed in the colon tissue from chronic colitis and UC patients and in the ileocecal junction from CD patients [81]. In contrast, mucosal T cells (in the lamina propria) from CD patients showed lower expression of BAX, proapoptotic protein, which indicate their resistance to apoptotic signals [82, 83]. This phenomenon was not observed in mucosal T cells from UC patients. Further studies confirmed that the mitochondria-driven apoptosis defect of mucosal T cells, thus abnormal T-cell mediated immune reaction in CD [84, 85]. However, it is not clear why levels of apoptosis differ between different tissue/cell types in CD.

Furthermore, the mitochondrial stress response, antioxidant defense mechanisms, such as heat-shock proteins 90 and 60, H^+^-transporting two-sector ATPase, prohibitin (PHB), mitochondrial malate dehydrogenase, voltage-dependent anion-selective channel protein 1, thioredoxin peroxidase, and thiol-specific antioxidants were also shown to be involved in UC [75]. The protein expression of PHB was also significantly decreased in disease-involved area of colonic mucosal biopsy samples from CD patients [86]. In an experimental setting, the functional consequence of PHB1 was confirmed using mice with intestinal epithelial cell-specific deletion of the *Phb1* gene. Both intestinal epithelial cell- and Paneth cell-specific deletion of the *Phb1* gene caused spontaneous ileitis characterized by mitochondrial dysfunction in mice [87]. The same study showed that treatment with the mitochondria-targeted antioxidant Mito-Tempo ameliorated the ileitis, suggesting the pathogenic role of mitochondrial ROS in CD. Other experimental evidence suggested the pathological involvement of mitochondrial ROS using mice deficient in the *Mdr1a* (multidrug resistance protein 1a) gene [59]. In the same study, these researchers showed that the experimental induction of mitochondrial ROS in the colon by treatment with rotenone (mitochondrial complex I-specific inhibitor) or deletion of the *Sod2* (superoxide dismutase 2, mitochondrial) gene exacerbated experimental colitis in mice and ameliorated colitis by treatment with a mitochondrial-specific antioxidant (e.g., MitoQ), confirming the role of mitochondrial ROS in colitis. As such, current knowledge of mitochondrial involvement in IBD is primarily mitochondrial ROS that are produced by intestinal cells and cause inflammation in the gut.

### Mitochondrial function in immune cells: immunometabolism

Development and activation of immune cells, another important cell type residing in the intestinal mucosa, are also finely regulated by mitochondrial metabolism. To meet their energy demand to function, immune cells reprogram their metabolic pathways. These metabolic pathways include glycolysis, the pentose phosphate pathway, the TCA cycle, FAO, FA synthesis, and amino acid metabolism and are thus heavily involved in mitochondrial functions. The metabolic status of immune cells largely impacts on their phenotype, such as pro- and anti-inflammatory status [88]. For T cells, mitochondrial ATP production during the first 24 to 48 h after T-cell activation is critical for full effector T-cell activation and proliferation [89]. An integrated study of the proteome and phosphoproteome during T-cell activation demonstrated that many mitochondrial metabolic pathways (e.g., mTORC1-dependent mitoribosome biogenesis and COX10-mediated complex IV activity) were altered, suggesting the critical involvement of mitochondrial metabolism in the initiation of T-cell activation [90]. In this line, impairment in OxPhos complex IV or the complex III subunit was shown to impact T-cell activation in vitro and in vivo [91–93]. Macrophages, another player in IBD, are known to polarize to either inflammatory M1 macrophages or anti-inflammatory M2 macrophages, depending on the immune response [94], and indeed, many M1 proinflammatory macrophages were observed in the gut of IBD patients [95]. Interestingly, mitochondrial metabolites from the TCA cycle, itaconate, and α-ketoglutarate are known to inhibit secretion of the proinflammatory cytokines IL-1 beta and IL-6 secretion and to increase IL-10 production when stimulated with lipopolysaccharide [96]. Nevertheless, whether dysregulations of immunometabolism in the intestinal mucosal immune cells are involved in IBD pathogenesis or whether those in the peripheral blood are observed in IBD patients need to be determined.

## Communication between gut microbiota and mitochondria in IBD

Both dysbiosis and mitochondrial dysfunction result in alteration in gut barrier function and immune cell activities, as discussed in the previous sections. In addition to their synergetic contribution to disease pathology, mitochondria and intestinal microbes are thought to interact via mediators, including endocrine, immune, and humoral links [97].

### Signals from gut microbiota to mitochondria: intestinal microbial metabolites

The most studied gut microbe-derived metabolites, SCFAs, are predominantly metabolized by enterocytes and the liver and are the energy source for tissue metabolism [98]. In brief, SCFAs are converted into acetyl-CoA, which is transferred to the TCA cycle in mitochondria, and further metabolic intermediates are generated. During this process, NADH^+^ and FADH^+^ are generated, fueled into the ETC, and utilized in ATP production. Butyrate inhibits glycolysis and switches cell metabolism toward gluconeogenic conditions, thus promoting lactate utilization [99]. Positive effects of SCFAs on the intestinal barrier have been reported. Treatment of human colon mucosal epithelial cells (NCM460) with sodium butyrate (SB) increased mitochondrial respiration (i.e., increased maximal respiration, spare capacity, and OxPhos-dependent ATP production) and expression of genes involved in mitochondrial bioenergetics (*TFAM*), complex I (*NDUFA1*, *NDUFA4*, and *NDUFA6*), complex IV (*COX6A1*), and complex V (*ATP5E* and *ATP8*). Additionally, SB treatment promoted barrier integrity by increasing tight junction gene (zonulin-1 and occludin) expression. These effects of SB were more pronounced when NCM460 cells were treated with TNF, suggesting the potential therapeutic potential of SB for inflammation-induced barrier disfunction [100]. Another recent study reported that microbially produced butyrate promoted intestinal homeostasis and that butyrate treatment in mice protected against colitis by repressing hexokinase 2 expression, which promoted epithelial cell death and impaired mitochondrial respiration [101]. SCFAs are also known to control the differentiation of CD4+ T cells ex vivo and in vivo [102–104]. More specifically, differentiation to Th1 cells was promoted, while that to Th17 cells was inhibited. Interestingly, treatment of Th1 and Th17 cells with SCFAs stimulated the production of the anti-inflammatory cytokine IL-10. The mechanism of this effect of SCFAs on CD4+ T cells involves regulation of mTOR (mammalian target of rapamycin; a master regulator of cell growth and metabolism) pathway. The increased production of ATP and the depletion of AMP activate mTOR pathway because ATP is an inhibitor, and AMP is an activator of AMPK, which has repressive activity against mTOR [105]. Additional explanation of this mechanism is that increased levels of acetyl-CoA by SCFAs promote the inhibition of histone deacetylase, resulting the elevation in acetylation of ribosomal protein S6 kinase beta-1, which is the downstream target of the mTOR pathway, thus leading to the activation of the pathway [105]. Additionally, the modulatory effects of SCFAs (primarily butyrate) on mitochondrial metabolism have been reported in macrophages, B cells, and innate lymphoid cells [105]. Thus, these experimental observations support that SCFAs are microbially derived mediators that modulate cellular metabolism in intestinal epithelial cells and immune cells by controlling mitochondrial function.

Large amounts of hydrogen sulfide (H_2_S) are also produced by the intestinal microbes via anaerobic metabolism [106]. At lower concentrations, H_2_S is cytoprotective against oxidative damage, increases sensitivity to several antibiotics, and increases resistance to the host immune response, while at higher concentrations, it is toxic [107]. To counteract excess bacterially derived exogenous H_2_S, host cells have a detoxifying mechanism [108]. H_2_S is oxidized to thiosulfate and sulfate by the mitochondria at the level of coenzyme Q (CoQ) through sulfide quinone oxidoreductase (SQR). During this oxidation by SQR, two electrons are released and transferred by flavin adenine dinucleotide to CoQ and are thus donated to the ETC stimulating mitochondrial bioenergetics [108]. As mentioned earlier, SCFAs are oxidized to acetyl-CoA, which is further utilized to generate ATP. Of note, butyrate is a major energy source in colonocytes, and H_2_S is known to inhibit beta-oxidation of butyrate [109]. As such, mitochondria in colonocytes process a limited amount of sulfide, which mitochondria are capable to detoxify this molecule, while they are able to recover energy [106]. Exogenous H_2_S was shown to control the substrate utilization from fatty acid oxidation to glucose oxidation in cardiomyocytes in mice [110]. These observations clearly indicate that H_2_S impacts mitochondrial metabolism, which further controls cellular metabolism defining cellular activities. Therefore, it is not surprising that H_2_S modulates the adaptive immune system, where differential immune subpopulations (e.g., Th1, Th2, Th17, and T regulatory cells) exhibit a well-defined metabolic profile [111, 112]. In the context of IBD, a previous study showed that increased sulfate-reducing bacteria, thereby increasing bacterially derived H_2_S levels, increased Th17- and T regulatory-cell-type cytokine production and activation profiles in mesenteric lymph nodes in experimental colitis [113]. This mechanism also applies to intestinal epithelial cells, supported by a report describing that microbially derived (and endogenous) H_2_S modulates colonocyte energy metabolism and is thus involved in pathophysiology in the gut [106].

Other examples of microbial metabolites that are known to modulate mitochondrial function are *p*-cresol and ammonia. *p*-cresol belongs to phenolic compounds, which are produced from aromatic amino acid L-tyrosine primarily anaerobic microbes in the large intestine [114]. When *p*-cresol presents in excess in colonocytes, it inhibits mitochondrial oxygen consumption and consequently reduces cell proliferation [115]. The same study also showed that when colonocytes were treated with *p*-cresol, anion superoxide production and DNA-double strand break were increased; thus, *p*-cresol is genotoxic. Ammonia is also produced by intestinal microbes and present at relatively high concentration in the colon. A recent study demonstrated that ammonia treatment on Caco-2 cells induced oxidative stress and mitochondrial dysfunction (e.g., reduced mitochondrial gene expression, reduction of TCA cycle intermediates, reduced mitochondrial membrane potential) [116]. At the same time, reduction of membrane localization of tight junction protein (ZO-1, ZO-2, occludin, claudin-1, and -3) and increased permeability in colonocytes were induced by the ammonia treatment. This study suggested that ammonia treatment triggered mitochondrial dysfunction causing the increase of oxidative stress, which was causal for intestinal barrier dysfunction.

### Signals from mitochondria to commensal bacteria

Mitochondria maintain homeostasis in the intestinal epithelial cells, and ROS are produced as byproduct of the OxPhos in the ETC, as mentioned earlier. The potential effect of mitochondrial ROS on gut microbiota is direct killing at higher concentrations [117]. This concept was supported by a study of mouse strains that produced differential levels of mitochondrial ROS, demonstrating the negative correlation between mitochondrial ROS levels and intestinal microbiota diversity [118].

Apart from exogenous bacterially derived H_2_S, host cells also endogenously produce H_2_S from the dietary amino acid methionine [108], and endogenous H_2_S controls cellular metabolism to maintain intestinal homeostasis together with exogenous H_2_S [106]. As discussed earlier, H_2_S is detoxified in the mitochondria. If any deficits in the mitochondrial H_2_S detoxification process exist, including genetic deficits in the SQR gene, H_2_S levels remain at toxic, which will cause tissue damage by modulating intestinal epithelial cellular metabolism, thus indirectly affecting the composition of the intestinal microbiota [119]. In fact, pathological relevance between mitochondrial H_2_S detoxification and IBD was reported: impaired H_2_S detoxification pathways were observed in colon biopsy samples obtained from CD patients. Moreover, the relative abundance of H_2_S-producing bacteria was increased in stool samples from CD patients [120]. In their study, whether mitochondrial dysfunction or intestinal dysbiosis is the primary cause of IBD was unclear.

### Mitochondrial DNA as a common modulator of mitochondrial function and the composition of the intestinal microbiota

Mitochondria have their own genome, called mitochondrial DNA (mtDNA). This small (approximately 16 kb in mammals) circular DNA encodes 37 genes: 13 protein coding, 2 rRNA, and 22 tRNA genes. mtDNA is polymorphic, and mutations/variations in mtDNA are causal not only for primary mitochondrial diseases, such as MELAS and LHON, but also for common complex diseases, including type 2 diabetes, Parkinson’s disease, and cancers [121]. For IBD, two studies, one from Germany and another from the UK, showed an association between mtDNA variants and UC [122, 123]. While the former reported that one specific polymorphism, 11719A > G, in the *MT-ND4* gene was associated with male patients affected with UC, the latter identified mtDNA variants with increased and/or decreased risks for UC. Interestingly, these UC candidate alleles are also risk factors or protective for other diseases, including schizophrenia, ankylosing spondylitis, ischemic stroke, Parkinson’s disease, and psoriasis [123]. This finding suggests that mtDNA variants modify the development of multiple late-onset diseases, including UC. There is experimental evidence reported that a mouse strain carrying *mt-Co3* (m.9348G > A), *mt-Tr* (m.9821ins. AA), and *mt-Nd3* (m.9461 T > C) (C57BL/6 J-^mtNOD/LtJ^) was significantly less susceptible to dextran sulfate sodium (DSS)–induced colitis, which is a murine experimental model for IBD, than wild-type C57BL/6 J mice [124]. In this study, the authors demonstrated that the functional consequence of these mtDNA variants was an increase in ATP production, thereby increasing the turnover of intestinal epithelial cells in the colon. Accordingly, this increased epithelial regeneration may be a protective effect against DSS–induced colitis by maintaining the intestinal barrier function. In contrast, another study demonstrated that the mucus layer of the colon in mice with a variant in the *mt-Atp8* gene (C57BL/6 J-mt^FVB/NJ^) had significantly less defective goblet cell differentiation than that of wild-type mice at the steady state [78]. These findings of differential observations (protective/susceptible) in mice carrying distinct mtDNA variants are in line with the abovementioned human study describing that mtDNA variants are both risk factors and protective in common diseases, including UC [123]. Although this mouse strain (C57BL/6 J-mt^FVB/NJ^) was not evaluated for DSS–induced colitis, a differential disease susceptibility compared with wild-type is expected. The mucus layer has a critical role in the interaction with gut microbiota by providing nutrients and attachment sites [125]; thus, changes in the mucus layer would directly impact the microbes in the gut. In fact, we found that the composition of the gut microbiota in C57BL/6 J-mt^FVB/NJ^ mice was significantly different from that in wild-type mice [126]. We also demonstrated that other mouse strains carrying distinct mtDNA variants (e.g., C57BL/6 J-mt^NZB/BlnJ^ carrying multiple mtDNA variants and C57BL/6 J-mt^129S1/SvlmJ^ carrying a variant in *mt-Cytb*) exhibited differential gut microbiota composition [126, 127]. With a differential gut microbiome, it is hypothesized that the profile of bacterial metabolites could be distinct between these mouse strains with differential mtDNA variants. Such changes may further result in the alteration of gut microbe-derived activation signals from the gut microbiota to intestinal immune cells and gut epithelial cells.

For the DSS-colitis-protective C57BL/6 J-mt^NOD/LtJ^ mice, the histological evaluation of the colon demonstrated that the lower inflammatory score in the C57BL/6 J-mt^NOD/LtJ^ mice with colitis compared with wild-type mice with colitis, and thus, evaluation of cellular metabolism in intestinal immune cells, including intestinal dendritic cells and CD4+ T cells, in these mouse strains carrying differential mtDNA variants at steady state and under stress is warranted. In fact, cellular metabolism, including the levels of OxPhos and glycolysis, was altered in quiescent CD4+ T cells from C57BL/6 J-mt^FVB/NJ^ mice compared with those from wild-type C57BL/6 J mice [126, 128]. This finding suggests that the immune cell response toward immunological stress, including bacterial metabolites, will be variable between mice carrying different mtDNA variants. Thus, again, profiling cellular metabolism in intestinal immune cells would confirm this hypothesis.

**Fig. 1:**
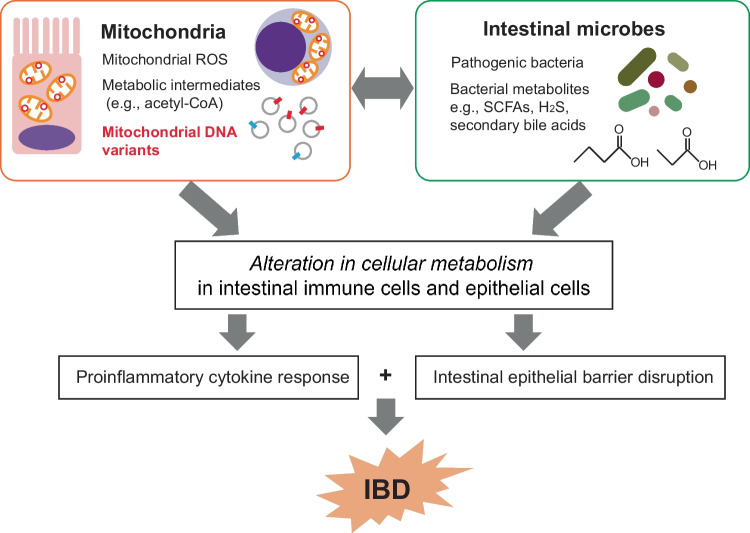
A schematic interaction between mitochondria and gut microbiota contributing to the disease pathology in IBD

Therefore, we propose that mtDNA variants are common modulators of pathological players (e.g., gut epithelial cells and immune cells, gut microbiome, and mitochondria) in IBD and thus could be a potential therapeutic target (Fig. 1).

## Current studies of therapeutic approaches to modulate both gut microbiota and mitochondrial function in IBD

Currently, several clinical studies to evaluate the therapeutic potential of modulations of gut microbiota or mitochondrial function for IBD patients are ongoing. To modulate the composition of gut microbiota, several clinical trials have been conducted by nutritional intervention in IBD (e.g., exclusive enteral nutrition, the CD/UC exclusion diet, the low fermentable oligosaccharides, disaccharides, monosaccharides, and polyols; FODMAP diet and the gluten-free diet) [129] or fecal microbiota transplantation in CD patients [130]. Alteration of the gut microbial composition will further result in changes in the quality and quantities of their metabolites; thus, signals from gut microbiota to mitochondria are expected to be changed. The use of probiotics and prebiotics is also of interest as a potential therapy for IBD, although further studies involving larger well-designed clinical trials are required [131]. This hypothesis is supported by a recent study demonstrating that treatment with probiotic consortia (individual and a mixture of *Lactobacillus* spp. and *Bifidobacterium* spp.) and their metabolites altered gut microbial composition and successfully ameliorated DSS–induced colitis [132]. In this line, not only the natural probiotics but also a genetically engineered probiotic, *E. coli* Nissle 1917 overexpressing the antioxidant genes catalase and superoxide dismutase, showed therapeutic efficacy in a DSS–induced colitis model [133]. The uniqueness of this approach is that this genetically engineered probiotic was initially designed to eliminate ROS and reduce inflammation in the gut, and it indeed succeeded in doing so. This specific probiotic was also able to alter the composition of the gut microbiota by significantly reducing the abundance of *Escherichia-Shigella*, which promotes IBD pathology. Similarly, treatment with nanoparticles containing the antioxidant astaxanthin was able to relieve the disease severity in DSS–induced colitis by inhibiting ROS production and mitochondrial depolarization as well as altering the composition of the gut microbiota [134]. Although the precise mechanism of these findings was not discussed, it is hypothesized that the scavenging (mitochondrial) ROS can possess dual effects on both intestinal inflammation and the gut microbial composition, which may be the secondary effect. As a clinical trial for the use of mitochondrial antioxidant substance (MitoQ) to treat UC has been undertaken [135], it will be worth to evaluate the gut microbe composition in this trial.

As discussed earlier, mtDNA variants would be a potential common regulator of both mitochondrial function and the intestinal microbiota. Therefore, modulating pathologically relevant mtDNA variants may be a potential approach to control IBD. Currently, there are several approaches to manipulate mtDNA, e.g., RNA-free programmable nucleases, including restriction enzymes [136, 137], transcription activator-like effector (TALE) nuclease [138], and zinc finger nuclease [139, 140], fused to mitochondrial targeting signal sequences to induce double-strand breaks in mtDNA. These approaches utilize protein-restricted nucleolysis to achieve a heteroplasmy shift. More recently, a novel technology based on the TALE system using bacterial deaminase (dsDNA deaminase toxin A; DddA) to specifically convert cytosines to thymines (C to T) called the DddA–derived cytosine base editor (DdCBE) system was reported [141]. More recently, the technique has been further developed and generated TALE-linked deaminase, which enables the conversion of adenine to guanine as well [142]. These novel tools are now able to mutagenize mtDNA in mice [143, 144]. Notably, the load of pathogenic mtDNA variants may vary across cells, tissues, and organs [145], and mtDNA editing should be designed accordingly. Therefore, mitochondrial genome editing may be the future option if causal mtDNA variants are identified in IBD patients, although further basic studies are needed.

## Conclusion and future prospects

Experimental and clinical studies conducted to date clearly indicate that mitochondrial functions in intestinal epithelial cells and immune cells together with gut microbiota and their metabolites contribute to the pathogenesis of IBD synergistically. Based on these findings, clinical trials and experimental studies (primarily using an experimental colitis mouse model) targeting either gut microbiota or mitochondria (primarily treatment with antioxidants to scavenge mitochondrial ROS) indeed lead to relief of the disease. These approaches (targeting either gut microbiota or mitochondrial ROS) were surprisingly able to modulate both the original therapeutic target and another, supporting the communication between gut microbiota and mitochondria. In addition to these therapeutic options under study, we propose that mtDNA variants relevant to IBD will be a novel target of treatment. One example to achieve this aim is the mitochondrial genome editing strategy, which has recently been rapidly advanced. Clinical application of such approaches is yet too early, requiring further basic studies including enabling organ- and cell-specific efficacy. This is a critical point because differential mucosal cell populations (e.g., epithelial cells, Paneth cells, intraepithelial lymphocytes, and macrophages) are the main players in the pathogenesis of IBD. Considering the increasing number of IBD patients and no availability of reliable treatment, such challenges and efforts to develop novel therapeutic options for IBD should be highly encouraged. In addition, such approaches will be further applied to many common complex diseases, which are not limited to gastroenterological disorders, but also to diseases including neurodegenerative diseases (e.g., Alzheimer’s disease) and metabolic diseases (obesity, type 2 diabetes, and cardiovascular disorders), as mtDNA variants are associated with such conditions, which affect majority of populations worldwide.

## Data Availability

Not applicable.

## Abbreviations

Acetyl-CoA: Acetyl coenzyme A
ATP: Adenosine triphosphate
IBD: Inflammatory bowel disease
CD: Crohn’s disease
ETC: Electron transport chain
H_2_S: Hydrogen sulfide
mtDNA: Mitochondrial DNA
OxPhos: Oxidative phosphorylation
ROS: Reactive oxygen species
SCFAs: Short chain fatty acids
UC: Ulcerative colitis
